# Increased healthcare costs by later stage cancer diagnosis

**DOI:** 10.1186/s12913-022-08457-6

**Published:** 2022-09-13

**Authors:** November McGarvey, Matthew Gitlin, Ela Fadli, Karen C. Chung

**Affiliations:** 1 BluePath Solutions, Los Angeles, CA USA; 2 GRAIL LLC, a subsidiary of Illumina Inc currently held separate from Illumina Inc under the terms of the Interim Measures Order of the European Commission dated 20 October 2021, 1525 O’Brien Dr, Menlo Park, CA 94025 USA

**Keywords:** Cancer, Stage, Diagnosis, Cost, Claims

## Abstract

**Background:**

Cancer represents a significant source of disease burden in the United States (US), both clinically and economically. Diagnosis and treatment of cancer at earlier stages may reduce this burden. To better understand potential impacts of earlier diagnosis, healthcare costs among patients with cancer were assessed by cancer type and stage at diagnosis.

**Methods:**

A retrospective analysis was conducted using Optum’s de-identified Integrated Claims-Clinical data set with Enriched Oncology, which includes data from Medicare Advantage and commercially insured members. Adult members newly diagnosed with solid tumor cancers, cancer stage at diagnosis (diagnosed 1/1/2016–6/30/2020), and continuous enrollment for at least one month post diagnosis were identified. Patients with breast, cervical, colorectal, lung, ovarian, or prostate cancer were reported. Mean standardized costs (2020 USD) were calculated in each month on an annual and cumulative basis through four years post-cancer diagnosis. In each month, costs were calculated for those with continuous enrollment and no death reported in the month. Mean annual cost per patient was estimated by summing month one to 12 mean costs and stratifying by stage at cancer diagnosis; annual year one to four costs were summed to determine cumulative costs.

**Results:**

Among members diagnosed 2016–2020 with breast, cervical, colorectal, lung, ovarian, or prostate cancer, 20,422 eligible members were identified. Mean costs increased by stage of diagnosis across all cancers at the annual and cumulative level through year four post diagnosis. Cumulative mean costs grew over time at a relatively similar rate across stages I to III and more dramatically in stage IV, except for cervical and lung cancer where the rate was relatively stable or slightly fluctuated across stages and ovarian cancer where stages III and IV both increased more sharply compared to stages I and II.

**Conclusions:**

Mean annual and cumulative healthcare costs through year four post cancer diagnosis were significantly higher among those diagnosed at later versus earlier cancer stages. The steeper increase in cumulative costs among those diagnosed in stage IV for many cancer types highlights the importance of earlier cancer diagnosis. Earlier cancer diagnosis may enable more efficient treatment, improve patient outcomes and reduce healthcare costs.

**Supplementary Information:**

The online version contains supplementary material available at 10.1186/s12913-022-08457-6.

## Background

Cancer represents a significant source of disease burden globally, and in the United States (US). In the US, estimates based on the National Cancer Institute’s (NCI) Surveillance Epidemiology and End Results (SEER) data suggest approximately one in two men and one in three women will develop invasive cancer within their lifetime [1], with a little over 1.9 million new cancer cases estimated in 2022 [2]. Furthermore, cancer is a leading cause of death worldwide [3] and the second leading cause of death in the US [4]. Cancer-related deaths were estimated at 609,360 in the US in 2022 [2]. Five-year survival rates for all cancers combined have increased substantially since the early 1960’s in the US (29 to 36 percentage points), with improvements likely due to treatment advances and earlier cancer diagnoses [2].

Identification and treatment of cancer at an early stage before it has a chance to spread or progress and require more complex and intensive treatment can meaningfully improve clinical outcomes, as well as help limit costs for cancer treatment and management [5]. Cancer is a large and growing source of economic burden with $183 billion in associated medical care costs estimated in the US in 2015 and projections based on population growth suggesting an increase to $246 billion by 2030 [6]. This may be an underestimation of the potential national expenditures in 2030, because this does not reflect that cost will likely increase as new, more expensive cancer treatments are developed and accepted as the standard of care. Developments in later-stage innovative oncology treatment are likely to drive this trend, as the later-stage oncology pipeline has increased by 77% from 2008 to 2018 [7].

Published evidence on the cost of cancer varies widely in methodology, and comprehensive cost data presented for multiple cancer types, stratified by stage, and over a timeframe greater than one year post diagnosis are limited [8–14]. This presents significant obstacles in attempts to estimate the cost differences and potential cost offsets of diagnosing cancer sooner and/or delaying progression. The objective of the following analysis is to estimate the costs of care among patients diagnosed with solid tumor cancer, by cancer type and stratified by stage, on an annual and cumulative basis from diagnosis through four years post diagnosis.

## Methods

A retrospective analysis was conducted using Optum’s de-identified Integrated Claims-Clinical dataset with Enriched Oncology [15–18], which included health record and medical and pharmacy claims data from Medicare Advantage and commercially insured members (January 1, 2008-July 31, 2020). This dataset documents patient care across varied provider and health care settings in the US for approximately 2.2 million patients with at least one solid tumor diagnosis and it has been widely used in research published in peer-reviewed publications [19]. Adult members identified with newly diagnosed solid tumor cancer including cancer staging data and having continuous enrollment for at least 30-days post diagnosis were included. This reporting of analysis results centers on those members diagnosed with six of the 18 solid tumor cancers assessed—breast, cervical, colorectal, lung, ovarian, or prostate cancer—between January 1, 2016 and June 30, 2020. These cancer types were chosen as the reporting focus due to their relatively large sample sizes by stage throughout the four years post diagnosis assessed. Although data were captured and assessed starting in January 2008, this analysis reports on those eligible patients diagnosed in the most recent time period covering 2016 to 2020 to highlight current costs and trends.

Patients were categorized into cancer types and stages at diagnosis based on enriched oncology data sourced from an Optum iterative natural language processing (NLP) development project using clinically-validated provider notes captured in electronic health records and linked by patient ID with claims data. The Optum NLP mines unstructured clinical notes using a supervised machine learning model that has been developed based on NLP scientist and clinical expert guidance and evaluated against an annotated test set [20]. Neoplasm type and histology indicated in the enriched oncology data were grouped into cancer types based on standard conventions and clinical recommendations (e.g., as reported by the National Institutes of Health (NIH), NCI SEER Program) (Additional Table 1). Stages were classified into numbered stages (I-IV), with I-III reflecting the presence of cancer, with the higher number indicating the larger the tumor and the more it has spread to nearby tissues, and IV indicating advanced, metastatic cancer that has spread to distant parts of the body, or via the American Joint Committee on Cancer (AJCC) TNM system in which the cancer is assigned a letter or number to describe the tumor (T), node (N), and metastasis (M) categories [21–23]. After consultation with oncology clinical experts, the TNM values for patients without number staging were converted as follows: any with M0 and N0 and T1 became stage I; any with M0 and N1/N2 and/or T2 became stage II; any with M0 and N3 and/or T3/T4 became stage III; and any with M1 became stage IV. Converting the staging data to a single system allowed for a larger sample size for analysis.

Upon identifying eligible patients with staging data in the clinical datasets with Enriched Oncology, claims data associated with these patients were evaluated to identify the earliest date of cancer diagnosis, also referred to as the disease index date. The index date was the date listed of the earliest claim in the medical records with a relevant International Classification of Diseases (ICD)-9-Clinical Modification (CM) or ICD-10-CM diagnosis code of the cancer type of interest. In cases where there was more than a 365-day (year) gap in claims with ICD-9/10 codes for cancer diagnosis, the claim date closest to the date of the cancer stage note was used. Pre-index and post-index periods of assessment, in reference to the index date, were constructed. The pre-index period included a fixed 6-months timeframe ending the day before the index date and was used to inform on clinical characteristics needed to calculate the Charlson Comorbidity Index (CCI). Patient demographics were identified as of the index date. The post-index period consisted of a variable timeframe with a minimum of one month after the index date that ended on the earliest of patient death, end of continuous enrollment (evaluated on a monthly basis), or the end of the study period (June 30, 2020). The post-index period was used to calculate healthcare costs and was assessed out as far as the end of year four after the index date.

Costs calculated in the analyses included total and cancer-specific costs. Total costs were defined as any costs among patients diagnosed with cancer, while cancer-specific costs, a subset of the total costs, required the presence of an ICD-9-CM or ICD-10-CM diagnosis code of the cancer type of interest, an ICD-9-CM or ICD-10-CM procedure code for radiation, or a cancer-related treatment National Drug Code (NDC) or Healthcare Common Procedure Coding System (HCPCs) code on the claim. Cancer-related treatments include antineoplastic agents, adjunctive therapies, and any other US Federal Drug Administration (FDA)-approved treatment for conditions caused by cancer or its treatment [24]. The costs utilized in this analysis reflects standardized costs calculated based on a proprietary Optum algorithm that reflects adjustment of allowable payment amounts sourced from the claim forms to estimate standardized costs that reduce potential local/regional or payer/plan differences across individual hospitals and providers and enable national normalization of costs for better comparison across patients, data sources, and geographic regions [25–27]. All dollar estimates were inflated to 2020 dollars using an Optum-provided inflation factor based on the Medical Care Component of the Consumer Price Index (CPI).

Population characteristics and annual and cumulative costs through year four post diagnosis were descriptively analyzed and reported on per standard formats for continuous and categorical variables and stratified by cancer type and stage. Mean standardized costs (2020 USD) were calculated in each month over a four-year timeframe post cancer diagnosis. Standard costs for eligible patients located in the claim tables were calculated by month and then added together for the time period of interest. Eligible patients for the cost calculations were those patients that met the study inclusion criteria and had continuous insurance coverage and no death recorded for the month being calculated. Standard costs ≥ $0 for the month being calculated were included. Costs were assumed to be $0 in the month(s) assessed for otherwise eligible members with no recorded claim to keep cost estimates conservative. Cost eligibility was considered on a monthly basis to help ensure the capture of most cancer patients despite variable follow-up post cancer diagnosis and to avoid the risk of bias by only including patients with a minimum follow-up period (i.e., selection bias to patients with better outcomes).

## Results

Among members diagnosed with breast, cervical, colorectal, lung, ovarian, and prostate cancer from 2016–2020, 20,422 eligible members were identified for inclusion in this analysis (breast cancer: 9,888 [48.4%]; cervical cancer: 1,866 [9.1%]; colorectal cancer: 2,407 [11.8%]; lung cancer: 3,459 [16.9%], ovarian cancer: 723 [3.5%]; prostate cancer: 2,079 [10.2%]) (Tables 1, 2, 3, 4, 5 and 6). Across all cancers assessed, the mean age ranged from 53.5 to 68.6 years. For members diagnosed with cancers not predominately or exclusively among females or males (colorectal and lung), the percentage female ranged from 46.0% to 59.5%. The primary insurance coverage types most common across all cancers were commercial (26.9% to 61.9%) and Medicare Advantage (23.1% to 64.8%). Most individuals resided in the Midwest (29.9% to 47.3%) or Northeast (26.0% to 63.0%) geographic regions. Mean CCI was below or equal to a score of 1.3 for all cancers, except for lung cancer which had scores that ranged from 1.8 to 2.2.

**Table 1:** Breast cancer member characteristics by stage, diagnosed 2016–2020 (*N* = 9,888)

Stage	**I**	**II**	**III**	**IV**
n (%)^a, b^	5,060 (51.2%)	3,373 (34.1%)	805 (8.1%)	650 (6.6%)
**Age**, mean (SD), years^b^	59.7 (11.5)	57.4 (12.4)	56.4 (12.8)	57.5 (12.9)
**Female Gender**, n (%)^b^	5,045 (99.7%)	3,348 (99.3%)	800 (99.4%)	644 (99.1%)
**Insurance Type**, n (%)^a, b^				
Commercial	2,865 (56.6%)	1,908 (56.6%)	462 (57.4%)	355 (54.6%)
Medicaid	200 (4.0%)	197 (5.8%)	48 (6.0%)	52 (8.0%)
Medicare Advantage	1,597 (31.6%)	954 (28.3%)	208 (25.8%)	180 (27.7%)
Multiple^c^	12 (0.2%)	5 (0.2%)	1 (0.1%)	2 (0.3%)
Unknown	386 (7.6%)	309 (9.2%)	86 (10.7%)	61 (9.4%)
**Geographic Region**, n (%)^a, b^				
Midwest	2,248 (44.4%)	1,527 (45.3%)	343 (42.6%)	254 (39.1%)
Northeast	1,876 (37.1%)	1,161 (34.4%)	255 (31.7%)	206 (31.7%)
South	382 (7.6%)	321 (9.5%)	96 (11.9%)	112 (17.2%)
West	440 (8.7%)	271 (8.0%)	77 (9.6%)	56 (8.6%)
Unknown	114 (2.3%)	93 (2.8%)	34 (4.2%)	22 (3.4%)
**CCI**, mean (SD)^d^	0.6 (1.1)	0.7 (1.2)	0.6 (1.2)	0.9 (1.4)
**CCI**, n (%)^a, d^				
0	1,639 (67.2%)	1,125 (66.1%)	254 (67.2%)	205 (58.2%)
1	438 (18.0%)	303 (17.8%)	71 (18.8%)	70 (19.9%)
2	199 (8.2%)	153 (9.0%)	26 (6.9%)	30 (8.5%)
≥ 3	164 (6.7%)	122 (7.2%)	27 (7.1%)	47 (13.4%)

Charlson Comorbidity Index; *SD* Standard deviation

^a^ Percentages may not total to 100% due to rounding

^b^ Demographics were calculated at the time of cancer diagnosis

^c^ Individuals had insurance eligibility from multiple types during this period

^d^ CCI was calculated among subjects with 6 months of continuous insurance eligibility prior to their cancer diagnosis

**Table 2:** Cervical cancer member characteristics by stage, diagnosed 2016–2020 (*N* = 1,866)

Stage	**I**	**II**	**III**	**IV**
n (%)^a, b^	1,300 (69.7%)	198 (10.6%)	215 (11.5%)	153 (8.2%)
**Age**, mean (SD), years^b^	58.6 (12.3)	57.5 (12.1)	60.4 (12.2)	59.9 (12.7)
**Female Gender**, n (%)^b^	1300 (100.0%)	198 (100.0%)	215 (100.0%)	153 (100.0%)
**Insurance Type**, n (%)^a, b^				
Commercial	97 (49.0%)	92 (42.8%)	73 (47.7%)	97 (49.0%)
Medicaid	27 (13.6%)	14 (6.5%)	14 (9.2%)	27 (13.6%)
Medicare Advantage	54 (27.3%)	82 (38.1%)	52 (34.0%)	54 (27.3%)
Multiple^c^	0 (0.0%)	1 (0.5%)	0 (0.0%)	0 (0.0%)
Unknown	20 (10.1%)	26 (12.1%)	14 (9.2%)	20 (10.1%)
**Geographic Region**, n (%)^a, b^				
Midwest	578 (44.5%)	87 (43.9%)	84 (39.1%)	65 (42.5%)
Northeast	484 (37.2%)	64 (32.3%)	76 (35.4%)	50 (32.7%)
South	114 (8.8%)	33 (16.7%)	32 (14.9%)	15 (9.8%)
West	104 (8.0%)	12 (6.1%)	18 (8.4%)	18 (11.8%)
Unknown	20 (1.5%)	2 (1.0%)	5 (2.3%)	5 (3.3%)
**CCI**, mean (SD)^d^	0.8 (1.2)	0.8 (1.3)	1.3 (1.7)	1.3 (1.6)
**CCI**, n (%)^a, d^				
0	447 (57.2%)	56 (57.7%)	42 (43.8%)	27 (36.5%)
1	192 (24.6%)	21 (21.6%)	21 (21.9%)	24 (32.4%)
2	81 (10.4%)	10 (10.3%)	13 (13.5%)	12 (16.2%)
≥ 3	62 (7.9%)	10 (10.3%)	20 (20.8%)	11 (14.9%)

Charlson Comorbidity Index; *SD* Standard deviation

^a^ Percentages may not total to 100% due to rounding

^b^ Demographics were calculated at the time of cancer diagnosis

^c^ Individuals had insurance eligibility from multiple types during this period

^d^ CCI was calculated among subjects with 6 months of continuous insurance eligibility prior to their cancer diagnosis

**Table 3:** Colorectal cancer member characteristics by stage, diagnosed 2016–2020 (*N* = 2,407)

Stage	**I**	**II**	**III**	**IV**
n (%)^a, b^	269 (11.2%)	581 (24.1%)	914 (38.0%)	643 (26.7%)
**Age**, mean (SD), years^b^	63.2 (12.5)	62.2 (13.8)	61.0 (13.0)	58.8 (13.2)
**Female Gender**, n (%)^b^	132 (49.1%)	267 (46.0%)	434 (47.5%)	298 (46.4%)
**Insurance Type**, n (%)^a, b^				
Commercial	126 (46.8%)	269 (46.3%)	456 (49.9%)	342 (53.2%)
Medicaid	7 (2.6%)	32 (5.5%)	53 (5.8%)	44 (6.8%)
Medicare Advantage	120 (44.6%)	255 (43.9%)	335 (36.7%)	201 (31.3%)
Multiple^c^	0 (0.0%)	1 (0.17%)	0 (0.0%)	0 (0.0%)
Unknown	16 (6.0%)	24 (4.1%)	70 (7.7%)	56 (8.7%)
**Geographic Region**, n (%)^a, b^				
Midwest	115 (42.8%)	244 (42.0%)	379 (41.5%)	269 (41.8%)
Northeast	83 (30.9%)	187 (32.2%)	313 (34.3%)	167 (26.0%)
South	42 (15.6%)	84 (14.5%)	128 (14.0%)	130 (20.2%)
West	21 (7.8%)	42 (7.2%)	57 (6.2%)	54 (8.4%)
Unknown	8 (3.0%)	24 (4.1%)	37 (4.1%)	23 (3.6%)
**CCI**, mean (SD)^d^	1.3 (1.6)	1.2 (1.7)	1.2 (1.7)	1.3 (1.6)
**CCI**, n (%)^a, d^				
0	70 (41.9%)	160 (47.9%)	243 (49.0%)	134 (37.1%)
1	46 (27.5%)	82 (24.6%)	112 (22.6%)	114 (31.6%)
2	23 (13.8%)	38 (11.4%)	58 (11.7%)	53 (14.7%)
≥ 3	28 (16.8%)	54 (16.2%)	83 (16.7%)	60 (16.6%)

Charlson Comorbidity Index; *SD* Standard deviation

^a^ Percentages may not total to 100% due to rounding

^b^ Demographics were calculated at the time of cancer diagnosis

^c^ Individuals had insurance eligibility from multiple types during this period

^d^ CCI was calculated among subjects with 6 months of continuous insurance eligibility prior to their cancer diagnosis

**Table 4:** Lung cancer member characteristics by stage, diagnosed 2016–2020 (*N* = 3,459)

Stage	**I**	**II**	**III**	**IV**
n (%)^a, b^	793 (22.9%)	483 (14.0%)	711 (20.6%)	1,472 (42.6%)
**Age**, mean (SD), years^b^	68.6 (9.9)	66.9 (10.1)	66.7 (9.9)	65.1 (10.6)
**Female Gender**, n (%)^b^	472 (59.5%)	246 (50.9%)	355 (49.9%)	746 (50.7%)
**Insurance Type**, n (%)^a, b^				
Commercial	213 (26.9%)	148 (30.6%)	202 (28.4%)	593 (40.3%)
Medicaid	34 (4.3%)	27 (5.6%)	51 (7.2%)	71 (4.8%)
Medicare Advantage	514 (64.8%)	285 (59.0%)	420 (59.1%)	721 (49.0%)
Multiple^c^	1 (0.1%)	0 (0.0%)	1 (0.1%)	5 (0.3%)
Unknown	31 (3.9%)	23 (4.8%)	37 (5.2%)	82 (5.6%)
**Geographic Region**, n (%)^a, b^				
Midwest	321 (40.5%)	203 (42.0%)	336 (47.3%)	575 (39.1%)
Northeast	301 (38.0%)	155 (32.1%)	200 (28.1%)	478 (32.5%)
South	101 (12.7%)	72 (14.9%)	114 (16.0%)	243 (16.5%)
West	46 (5.8%)	30 (6.2%)	45 (6.3%)	137 (9.3%)
Unknown	24 (3.0%)	23 (4.8%)	16 (2.3%)	39 (2.7%)
**CCI**, mean (SD)^d^	2.2 (1.9)	2.0 (1.9)	2.0 (1.8)	1.8 (1.8)
**CCI**, n (%)^a, d^				
0	66 (16.5%)	58 (21.1%)	73 (17.8%)	219 (24.1%)
1	116 (28.9%)	92 (33.5%)	128 (31.2%)	275 (30.3%)
2	80 (20.0%)	41 (14.9%)	79 (19.3%)	173 (19.1%)
≥ 3	139 (34.7%)	84 (30.5%)	130 (31.7%)	240 (26.5%)

Charlson Comorbidity Index; *SD* Standard deviation

^a^ Percentages may not total to 100% due to rounding

^b^ Demographics were calculated at the time of cancer diagnosis

^c^ Individuals had insurance eligibility from multiple types during this period

^d^ CCI was calculated among subjects with 6 months of continuous insurance eligibility prior to their cancer diagnosis

**Table 5:** Ovarian cancer member characteristics by stage, diagnosed 2016–2020 (*N* = 723)

Stage	**I**	**II**	**III**	**IV**
n (%)^a, b^	247 (34.2%)	79 (10.9%)	270 (37.3%)	127 (17.6%)
**Age**, mean (SD), years^b^	53.5 (14.6)	58.5 (12.1)	59.6 (13.3)	61.5 (11.3)
**Female Gender**, n (%)^b^	247 (100.0%)	79 (100.0%)	270 (100.0%)	127 (100.0%)
**Insurance Type**, n (%)^a, b^				
Commercial	153 (61.9%)	46 (58.2%)	131 (48.5%)	71 (55.9%)
Medicaid	15 (6.1%)	8 (10.1%)	10 (3.7%)	4 (3.2%)
Medicare Advantage	57 (23.1%)	24 (30.4%)	99 (36.7%)	46 (36.2%)
Multiple^c^	0 (0.0%)	0 (0.0%)	2 (0.7%)	0 (0.0%)
Unknown	22 (8.9%)	1 (1.3%)	28 (10.4%)	6 (4.7%)
**Geographic Region**, n (%)^a, b^				
Midwest	105 (42.5%)	25 (31.7%)	115 (42.6%)	45 (35.4%)
Northeast	92 (37.3%)	27 (34.2%)	78 (28.9%)	34 (26.8%)
South	26 (10.5%)	15 (19.0%)	47 (17.4%)	23 (18.1%)
West	18 (7.3%)	8 (10.1%)	26 (9.6%)	17 (13.4%)
Unknown	6 (2.4%)	4 (5.1%)	4 (1.5%)	8 (6.3%)
**CCI**, mean (SD)^d^	0.7 (1.1)	0.9 (1.4)	1.0 (1.5)	1.2 (1.6)
**CCI**, n (%)^a, d^				
0	73 (64.0%)	20 (57.1%)	68 (57.6%)	32 (44.4%)
1	23 (20.2%)	8 (22.9%)	21 (17.8%)	16 (22.2%)
2	10 (8.8%)	4 (11.4%)	14 (11.9%)	14 (19.4%)
≥ 3	8 (7.0%)	3 (8.6%)	15 (12.7%)	10 (13.9%)

Charlson Comorbidity Index; *SD* Standard deviation

^a^ Percentages may not total to 100% due to rounding

^b^ Demographics were calculated at the time of cancer diagnosis

^c^ Individuals had insurance eligibility from multiple types during this period

^d^ CCI was calculated among subjects with 6 months of continuous insurance eligibility prior to their cancer diagnosis

**Table 6:** Prostate cancer member characteristics by stage, diagnosed 2016–2020 (*N* = 2,079)

Stage	**I**	**II**	**III**	**IV**
n (%)^a, b^	459 (22.1%)	815 (39.2%)	264 (12.7%)	541 (26.0%)
**Age**, mean (SD), years^b^	62.8 (8.0)	66.2 (8.3)	65.8 (9.6)	68.1 (10.4)
**Female Gender**, n (%)^b^	0. (0.0%)	0 (0.0%)	0 (0.0%)	0 (0.0%)
**Insurance Type**, n (%)^a, b^				
Commercial	273 (59.5%)	344 (42.2%)	107 (40.5%)	214 (39.6%)
Medicaid	11 (2.4%)	24 (2.9%)	6 (2.3%)	30 (5.6%)
Medicare Advantage	156 (34.0%)	383 (47.0%)	127 (48.1%)	264 (48.8%)
Multiple^c^	0 (0.0%)	2 (0.3%)	1 (0.4%)	1 (0.2%)
Unknown	19 (4.1%)	62 (7.6%)	23 (8.7%)	32 (5.9%)
**Geographic Region**, n (%)^a, b^				
Midwest	137 (29.9%)	411 (50.4%)	116 (43.9%)	216 (39.9%)
Northeast	289 (63.0%)	290 (35.6%)	101 (38.3%)	195 (36.0%)
South	20 (4.4%)	57 (7.0%)	31 (11.7%)	61 (11.3%)
West	7 (1.5%)	38 (4.7%)	8 (3.0%)	49 (9.0%)
Unknown	6 (1.3%)	19 (2.3%)	8 (3.0%)	20 (3.7%)
**CCI**, mean (SD)^d^	0.7 (1.2)	0.9 (1.4)	1.3 (1.8)	1.2 (1.8)
**CCI**, n (%)^a, d^				
0	190 (66.0%)	269 (56.4%)	77 (50.3%)	148 (44.8%)
1	54 (18.8%)	109 (22.9%)	26 (17.0%)	88 (26.7%)
2	24 (8.3%)	49 (10.3%)	18 (11.8%)	43 (13.0%)
≥ 3	20 (6.9%)	50 (10.5%)	32 (20.9%)	51 (15.5%)

Charlson Comorbidity Index; *SD* Standard deviation

^a^ Percentages may not total to 100% due to rounding

^b^ Demographics were calculated at the time of cancer diagnosis

^c^ Individuals had insurance eligibility from multiple types during this period

^d^ CCI was calculated among subjects with 6 months of continuous insurance eligibility prior to their cancer diagnosis

### Total costs and trends among patients with cancer

**Fig. 1:**
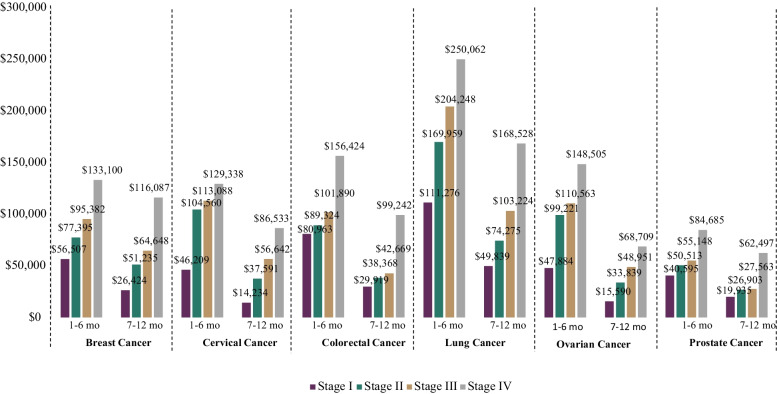
Mean cost by cancer and stage, year 1 post diagnosis

**Fig. 2:**
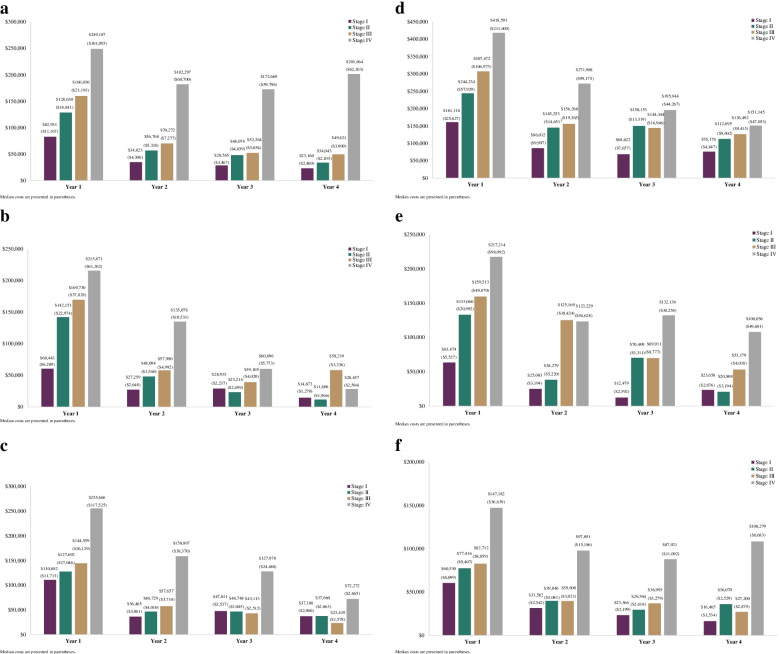
**a**. Breast cancer mean cost by stage at diagnosis, year 1–4 post diagnosis **b**. Cervical cancer mean cost by stage at diagnosis, year 1–4 post diagnosis **c**. Colorectal cancer mean cost by stage at diagnosis, year 1–4 post diagnosis **d**. Lung cancer mean cost by stage at diagnosis, year 1–4 post diagnosis **e**. Ovarian cancer mean cost by stage at diagnosis, year 1–4 post diagnosis **f**. Prostate cancer mean cost by stage at diagnosis, year 1–4 post diagnosis

Mean standard costs for cancer patients demonstrated consistent trends by stage and time post diagnosis across all cancers (Figs. 1 and 2a-f). In the first year post diagnosis, mean costs increased by stage and were higher in the first six months as compared to the second half of the year across all cancers and stages. Mean costs increased in the first half of the year compared to the second half across stages 1.1 to 2.1 times for breast cancer; 1.5 to 3.2 times for cervical cancer; 1.6 to 2.7 times for colorectal cancer; 1.5 to 2.3 times for lung cancer; 2.2 to 3.1 times for ovarian cancer, and 1.4 to 2.0 times for prostate cancer.

At the annual and cumulative level, mean costs generally increased by stage of diagnosis across cancer types (Figs. 2a-f and Additional Figs. 1a-f). A handful of fluctuations were noted in years 3 and/or 4 for some cancers, which are likely reflective of limited sample sizes in later years and a wide range of cost values. However, in each year assessed for all cancers, stage IV costs were consistently higher than stage I costs. In comparing years 1 to 4, the majority of costs were incurred in the first year representing between 30.9% to 63.2% of total cumulative costs across cancers and stages. The cumulative mean costs grew over time from year 1 to 4 at a relatively similar rate across stages I to III and more dramatically in stage IV, except for cervical and lung cancer where the rate of increase was relatively stable or fluctuated across stages and ovarian cancer where stages III and IV both increased more sharply compared to stages I and II.

As expected, standard deviations were positively skewed and relatively large compared to the standard cost means as well as, with a few exceptions, increasing in value by stage (Additional Table 2). This trend is likely reflective of wide variation in healthcare resource use by patients with cancer. Standard cost by stage for the other 12 cancers assessed but not included in the main results are also available in Additional Table 2.

### Cancer-specific costs and trends among patients with cancer

When examining the subset of cancer-specific claims from the total costs in each year post-diagnosis, the cancer-specific costs represented a meaningful proportion of the total costs across cancer types and stages (year 1: 59.0% to 87.6%; year 2: 14.9% to 86.8%; year 3: 19.8% to 85.4%; year 4: 16.4% to 91.5%) (Additional Figs. 2 and 3a-f). The proportion of cancer-specific costs of the year 1 total were consistently large across cancer types and stages assessed. For stages I-III across cancer types, the percentage contribution of cancer-specific costs to the overall costs generally dropped in year 2 and held relatively constant or further decreased through year 4. The percentage contribution of cancer specific costs to the total for stage IV was less consistent, but generally remained high or even sometimes increased by year 4.

Similar trends in annual and cumulative mean cancer-specific costs increasing by stage were observed across cancer types (Additional Figs. 3a-f and 4a-f). Additionally, the majority of cancer-specific costs occurred in the first year, representing between 31.9% to 73.0% of total mean cumulative costs through year 4. In line with the total costs, the cumulative mean cancer-specific costs increased from year 1 to 4 at a relatively similar rate across stages I to III and more steeply in stage IV, except for cervical and lung cancer where the rate of increase is similar or varied across stages and ovarian cancer where stages III and IV increased more sharply than stages I and II.

## Discussion

The results of this analysis help address the large gap in evidence on US healthcare cost by stage of cancer with an assessment of multiple cancer types and through a timeframe covering up to four years post cancer diagnosis. Comparisons to published literature are challenging: few provide cost data by stage at diagnosis; some are specific to certain treatments; others report on a mean per patient per month and/or treatment phase basis not reflecting individual differences in costs by time post diagnosis, and some reflect less current data and differing types of costs and insurance-coverage population mixes (e.g., Medicare fee for service only or private insurance population; claims with paid amounts; and chart reviews with charged amounts). Publications on cost data are also often limited to the more common cancer types (e.g., breast, colorectal, lung). The few US studies that report by stage or other representation of stage (e.g., metastatic with no progression versus metastatic with progression) showed similar trends in that the costs for patients diagnosed at a later, more advanced, metastatic, or progressed stage were considerably higher than those diagnosed at an earlier, non-metastatic, or non-progressed stage [12, 14, 28–30]. Increasing costs by stage were also depicted in a model [31] which combined published US cost estimates by stage with incidence rates by stage at diagnosis. Several ex-US studies also support these findings [29, 32, 33].

Stage, age, and gender distribution by cancer type in this study was compared to national data accessed from the United States Cancer Statistics (USCS) data visualization tool produced by the Centers for Disease Control and Prevention (CDC) and the National Cancer Institute (NCI) which sources data from the CDC’s National Program of Cancer Registries (NPCR) and the NCI’s Surveillance, Epidemiology, and End Results (SEER) Program [34]. In general, trends in distributions by stage, age, and gender were relatively consistent between the study and USCS data with some deviations likely a reflection of the commercially insured population captured in this study (Additional Tables 3a-f).

Cost results for the first year post diagnosis in this study were generally higher than those described in other published data presented by stage [12, 28–30, 35, 36] and represent a mixture of stages [10]. However, this may be reflective of differing data sources, time periods, populations, included costs (e.g., cancer-specific costs, paid amounts, standardized costs), and inclusion of earlier years of data. Additionally, studies that restricted analyses to patients with a full year of data post diagnosis may result in bias with regard to selection of healthier patients that may be less costly. Similarly, requiring continuous insurance coverage for the entire year or timeframe assessed in some studies likely biased them towards selection of those that received better, uninterrupted care and management of their cancer. These types of patients may have been able to avoid potential costs related to delays in treatment and disease progression. The imputation of $0 values in the current analyses for patients otherwise eligible in months where a claim was not present helped ensure that the results did not overestimate mean annual costs. Additionally, this study did not take into consideration the cost of patients’ end-of-life care. Patients diagnosed with late stage cancer may survive less than one year and/or have high end of life care costs compared to patients with diagnosis at early stage cancer that may survive for many years and not be subject to these costs as well as potentially having lower costs in relation to less intensive cancer-related care needed.

In order to further evaluate the potential association between cost and stage at diagnosis and provide support to descriptive findings, a generalized linear model regression of mean monthly year 1 cost and stage at diagnosis was run by cancer type that adjusted for key patient and other characteristics. Characteristics included CCI, geographic region, race/ethnicity, gender, age, insurance coverage type, and the month the cost data was captured in. After adjusting for these characteristics, results confirmed that mean monthly costs in year 1 were substantially (additional $4,916 to $19,036) and statistically significantly (*p* < 0.0001) higher among patients diagnosed at stage IV versus stage I (Additional Table 4).

Similar to other studies that analyzed costs among cancer patients beyond the first year post diagnosis [10, 14, 28, 30], this analysis found that the bulk of costs occurred in the first year post diagnosis and then generally decreased in the second year and subsequently held relatively constant or slightly decreased through later years. Cumulative costs through year four also displayed similar trends of increase by stage as annual costs, with the steepest increases in cost among those diagnosed with cancer in stage IV for many cancer types. Thus, a cancer diagnosis, especially at a later, more advanced stage, may signify significant economic burden to payers that may extend throughout multiple years. This analysis was limited to a four year post-diagnosis time period, however, a longer timeframe may reveal a continued increase in healthcare costs, as well as capture costs related to recurrence and relapse. Beyond associated clinical benefits, reducing the proportion of the population with later-stage cancer diagnoses, especially in stage IV, may limit the need for more intensive and expensive treatments, increase patient’s health-related quality of life, have a significant impact in managing healthcare costs among cancer patients, and reduce caregiver and societal burden.

The limitations of this study include those inherent in any retrospective analysis. This study was limited to those individuals with commercial or private Medicare Advantage health coverage. Consequently, results of this analysis may not be generalizable to patients with other insurance or without health insurance coverage. Direct costs represented in these data reflected the standardized cost which may not reflect actual costs or paid amounts from adjudicated claims or demonstrate differences in these costs by individual hospital, provider, or insurance coverage type. Furthermore, the cancer-specific cost subgroup analysis relied on the accuracy of claims (inclusion of a cancer diagnosis code) to only capture cancer-specific costs. If unrelated costs were included in a claim where a cancer diagnosis was recorded, this may have overestimated cancer-specific costs. Additionally, as there is no standardized methodology for cancer staging within Optum, groupings were limited to the accuracy of staging as noted in the electronic medical record. However, staging was verified by Optum’s proprietary algorithm using NLP.

## Conclusion

This comprehensive analysis of multiple cancer types demonstrates that mean annual and cumulative costs of care per patient during the first four years post cancer diagnosis were significantly higher among those diagnosed at later versus earlier cancer stages. While healthcare costs were highest in the first-year post diagnosis, meaningful cost amounts were sustained throughout the end of year four post diagnosis and the majority of these costs were recorded as being cancer-specific. The steeper rate in increase in mean cumulative costs among those diagnosed in stage IV underscores the importance of diagnosing cancer as early as possible before metastasis. Earlier cancer diagnosis may enable more efficient treatment, improve patient outcomes, avoid complications and disease progression and reduce healthcare resource utilization and associated costs.

## Supplementary Information

**Additional file 1: Additional Table 1. **Rules for groupings into cancer types based on neoplasm and histology. **Additional Table 2. **Total cost by cancer type and stage, year 1 post diagnosis, diagnosed 2016-2020. **Additional Table 3a. **Breast cancer member characteristics by stage, diagnosed 2016-2020 comparison with USCS data as of 2019. **Additional Table 3b. **Cervical cancer member characteristics by stage, diagnosed 2016-2020 comparison with USCS data as of 2019. **Additional Table 3c. **Colorectal cancer member characteristics by stage, diagnosed 2016-2020 comparison with USCS data as of 2019. **Additional Table 3d. **Lung cancer member characteristics by stage, diagnosed 2016-2020 comparison with USCS data as of 2019. **Additional Table 3e. **Ovarian cancer member characteristics by stage, diagnosed 2016-2020 comparison with USCS data as of 2019. **Additional Table 3f. **Prostate cancer member characteristics by stage, diagnosed 2016-2020 comparison with USCS data as of 2019. **Additional Table 4. **Generalized linear regression analysis on monthly treatment costs during year 1 by stage at diagnosis. **Additional Figure 1a. **Breast cancer mean cost by stage at diagnosis, cumulative through year 4 post diagnosis. **Additional Figure 1b. **Cervical cancer mean cost by stage at diagnosis, cumulative through year 4 post diagnosis. **Additional Figure 1c. **Colorectal cancer mean cost by stage at diagnosis, cumulative through year 4 post diagnosis. **Additional Figure 1d. **Lung cancer mean cost by stage at diagnosis, cumulative through year 4 post diagnosis. **Additional Figure 1e. **Ovarian cancer mean cost by stage at diagnosis, cumulative through year 4 post diagnosis. **Additional Figure 1f**. Prostate cancer mean cost by stage at diagnosis, cumulative through year 4 post diagnosis. **Additional Figure 2**. Mean cancer-specific cost by cancer and stage, year 1 post diagnosis: 1-6 months and 7-12 months. **Additional Figure 3a. **Breast cancer mean cancer-specific cost by stage at diagnosis, year 1-4 post diagnosis. **Additional Figure 3b. **Cervical cancer mean cancer-specific cost by stage at diagnosis, year 1-4 post diagnosis. **Additional Figure 3c. **Colorectal cancer mean cancer-specific cost by stage at diagnosis, year 1-4 post diagnosis. **Additional Figure 3d. **Lung cancer mean cancer-specific cost by stage at diagnosis, year 1-4 post diagnosis. **Additional Figure 3e. **Ovarian cancer mean cancer-specific cost by stage at diagnosis, year 1-4 post diagnosis. **Additional Figure 3f. **Prostate cancer mean cancer-specific cost by stage at diagnosis, year 1-4 post diagnosis. **Additional Figure 4a. **Breast cancer mean cancer-specific cost by stage at diagnosis, cumulative through year 4 post diagnosis. **Additional Figure 4b. **Cervical cancer mean cancer-specific cost by stage at diagnosis, cumulative through year 4 post diagnosis. **Additional Figure 4c. **Colorectal cancer mean cancer-specific cost by stage at diagnosis, cumulative through year 4 post diagnosis. **Additional Figure 4d. **Lung cancer mean cancer-specific cost by stage at diagnosis, cumulative through year 4 post diagnosis. **Additional Figure 4e. **Ovarian cancer mean cancer-specific cost by stage at diagnosis, cumulative through year 4 post diagnosis. **Additional Figure 4f**. Prostate cancer mean cancer-specific cost by stage at diagnosis, cumulative through year 4 post diagnosis.

## Data Availability

The datasets generated and analyzed during the current study are proprietary to Optum and not publicly available due to legal restrictions. Researchers will fulfill reasonable requests for supplementary materials or information not subject to legal restrictions; requests can be made to the corresponding author, Karen C. Chung. An independent research group could access the data to replicate the study by contacting Optum (contact information below) to contract with them to purchase the Integrated Claims-Clinical dataset and the Enriched Oncology dataset for a specified length of access and covering a matching timeframe and population for the analysis in question. Data use, security, and transfer would need to be documented and agreed upon by Optum and the independent research group. Optum contact information. Phone: 1–866-306–1321. Email: connected@optum.com. Website: optum.com/life-sciences-solutions.

## Abbreviations

AJCC: American Joint Committee on Cancer
CCI: Charlson comorbidity index
CDC: Centers for Disease Control and Prevention
CPI: Consumer price index
FDA: Food and Drug Administration
HCPCS: Healthcare Common Procedure Coding System
ICD-9-CM: International Classification of Diseases, Ninth Revision, Clinical Modification
ICD-10-CM: International Classification of Diseases, Tenth Revision, Clinical Modification
NCCN: National Comprehensive Cancer Network
NCI: National Cancer Institute
NDC: National Drug Code
NIH: National Institutes of Health
NLP: Natural language processing
NPCR: National Program of Cancer Registries
SEER: Surveillance, Epidemiology, and End Results
TNM: Tumor (T), node (N), and metastasis (M)
US: United States
USCS: United States Cancer Statistics
