# Supplementary material for: Increased healthcare costs by later stage cancer diagnosis
Source: BMC Health Serv Res. 2022 Sep 13;22:1155. doi: 10.1186/s12913-022-08457-6 (PMC9469540; doi:10.1186/s12913-022-08457-6)
Supplement: Supplementary file 1 — Additional file 1: Additional Table 1. Rules for groupings into cancer types based on neoplasm and histology. Additional Table 2. Total cost by cancer type and stage, year 1 post diagnosis, diagnosed 2016-2020. Additional Table 3a. Breast cancer member characteristics by stage, diagnosed 2016-2020 comparison with USCS data as of 2019. Additional Table 3b. Cervical cancer member characteristics by stage, diagnosed 2016-2020 comparison with USCS data as of 2019. Additional Table 3c. Colorectal cancer member characteristics by stage, diagnosed 2016-2020 comparison with USCS data as of 2019. Additional Table 3d. Lung cancer member characteristics by stage, diagnosed 2016-2020 comparison with USCS data as of 2019. Additional Table 3e. Ovarian cancer member characteristics by stage, diagnosed 2016-2020 comparison with USCS data as of 2019. Additional Table 3f. Prostate cancer member characteristics by stage, diagnosed 2016-2020 comparison with USCS data as of 2019. Additional Table 4. Generalized linear regression analysis on monthly treatment costs during year 1 by stage at diagnosis. Additional Figure 1a. Breast cancer mean cost by stage at diagnosis, cumulative through year 4 post diagnosis. Additional Figure 1b. Cervical cancer mean cost by stage at diagnosis, cumulative through year 4 post diagnosis. Additional Figure 1c. Colorectal cancer mean cost by stage at diagnosis, cumulative through year 4 post diagnosis. Additional Figure 1d. Lung cancer mean cost by stage at diagnosis, cumulative through year 4 post diagnosis. Additional Figure 1e. Ovarian cancer mean cost by stage at diagnosis, cumulative through year 4 post diagnosis. Additional Figure 1f. Prostate cancer mean cost by stage at diagnosis, cumulative through year 4 post diagnosis. Additional Figure 2. Mean cancer-specific cost by cancer and stage, year 1 post diagnosis: 1-6 months and 7-12 months. Additional Figure 3a. Breast cancer mean cancer-specific cost by stage at diagnosis, year 1-4 post diagnosis. A [file 12913_2022_8457_MOESM1_ESM.docx]

**Additional Files**

**Additional Table 1. Rules for Groupings into Cancer Types Based on Neoplasm and Histology**

| Cancer Grouping Names | Neoplasm Types Included^a^ | Histology Types Included |
| --- | --- | --- |
| Breast | | |
| Breast | Mammary | No restrictions |
| Digestive System | | |
| Anal | Anal canal  Anorectal  Anus | No restrictions |
| Colorectal | Ascending colon  Cecum  Colon  Colorectal  Descending colon  Sigmoid  Rectal  Rectosigmoid junction  Transverse colon | No restrictions |
| Esophagus | Cervical esophagus  Esophageal  Esophagogastric junction  Gastroesophageal cancer | No restrictions |
| Pancreas | Head of Pancreas  Pancreatic  Pancreatic duct | No restrictions |
| Stomach | Cardia of stomach  Omentum  Stomach | No restrictions |
| Endocrine System | | |
| Thyroid and other endocrine | Thyroid gland  Adrenal gland | No restrictions |
| Genital Organs | | |
| Cervix (uterus) | Body of uterus  Endocervix  Endometrial  Uterine | No restrictions |
| Ovary | Ovarian | No restrictions |
| Prostate | Prostatic | No restrictions |
| Testis | Testicular | No restrictions |
| Vagina, vulva and other genital organs, female | Vagina and other genital organs, female   - Fallopian tubes - Vaginal   Vulva   - Vulvar | No restrictions |
| Head and Neck | | |
| Head and Neck | Larynx   - Laryngeal   Oral cavity and the pharynx   - Cheek* (lining) - Epiglottis - Floor of mouth - Glottis - Gums (behind wisdom teeth) - Hard palate - Hypopharyngeal - Lip* (lining) - Mouth* (floor of mouth under tongue) - Oropharyngeal - Nasopharyngeal - Parotid gland - Pharyngeal/pharynx - Pharyngeal wall (part of hypopharynx) - Postcricooid area - Pyriform Sinus (part of hypopharynx) - Salivary duct - Salivary duct gland - Salivary gland - Supraglottis - Tongue - Tonsillar   Other head and neck   - Ethmoidal sinus - Maxillary sinus - Nasal cavity - Paranasal sinus - Vocal cord | No restrictions except those marked with * which should only be included if do not have a + melanoma or + merkel cell or + squamous or + basal or + sarcoma in histology name |
| Respiratory System | | |
| Lung and bronchus | Bronchial  Bronchogenic  Lung | No restrictions |
| Skin (excluding basal and squamous) | | |
| Melanoma of the skin and non-epithelial skin | Arm  Abdominal  Back  Chin  Cheek  Chest wall  Ear  Facial  Femoral  Forearm  Forehead  Foot  Hand  Head and neck  Helix of ear  Jaw  Leg  Lip  Mandibular  Neck  Nose  Pelvic  Skin  Skin of Scalp  Tibia  Thigh  Shoulder | + Melanoma or + Merkel cell in histology name; all others exclude |
| Urinary System | | |
| Kidney and renal pelvis | Kidney  Renal pelvis  Wilms tumor | No restrictions |
| Ureter, urinary bladder (+urothelial) and other urinary organs | Ureter and other urinary organs   - Ureteral - Urethral   Urinary bladder (+urothelial)   - Bladder - Urinary bladder neck - Urothelial | No restrictions |
| All Other Sites | | |
| Other | Axilla  Bone  Brain  Eye  Eyelid  Other digestive   - Ampulla of vater - Appendix - Gastrointestinal   Gallbladder and other biliary   - Gallbladder   Liver and intrahepatic bile duct   - Bile duct - Common bile duct - Liver   Small intestine   - Duodenal - Intestine - Small intestine   Penis and other genital organs, male   - Penile - Peritoneal - Retroperitoneal   Soft tissue   - Arm - Abdominal - Back - Chin - Cheek - Chest wall - Ear - Facial - Femoral - Forearm - Forehead - Foot - Hand - Head and neck - Helix of ear - Jaw - Leg - Lip - Mandibular - Neck - Nose - Pelvic - Skin - Skin of scalp - Tibia - Thigh - Shoulder   Thymus | Not to include + basal or + squamous in the histology name  Re: Soft Tissue: + Sarcoma in histology name; all others exclude |

^a^ Text in grey italics were not specific neoplasm types listed in the data. They are included to show conceptual groupings.

**Additional Table 2. Total Cost by Cancer Type and Stage, Year 1 Post Diagnosis, Diagnosed 2016-2020**

| Stage | **I** | **II** | **III** | **IV** |
| --- | --- | --- | --- | --- |
| **Anal Cancer** | N=160 | | | |
| n | 24 | 60 | 60 | 16 |
| Standard Cost, mean (SD) | $105,141 ($66,683) | $108,373 ($80,759) | $246,086 ($200,720) | $122,868 ($76,985) |
| Standard Cost, median (range) | $41,175 ($110 - $719,735) | $26,679 ($268 - $1,287,678) | $79,500 ($170 - $3,036,147) | $42,808 ($478 - $661,521) |
| **Breast Cancer** | N=9,888 | | | |
| n | 5,060 | 3,373 | 805 | 650 |
| Standard Cost, mean (SD) | $82,931 ($66,159) | $128,630 ($96,277) | $160,030 ($101,381) | $249,187 ($146,306) |
| Standard Cost, median (range) | $11,167 ($3 - $4,802,518) | $14,841 ($12 - $7,377,044) | $21,193 ($11 - $3,610,989) | $101,055 ($93 - $5,741,402) |
| **Cervical Cancer** | N=1,866 | | | |
| n | 1300 | 198 | 215 | 153 |
| Standard Cost, mean (SD) | $60,443 ($54,758) | $142,151 ($96,193) | $169,730 ($105,101) | $215,871 ($134,889) |
| Standard Cost, median (range) | $6,289 ($31 - $2,374,156) | $22,974 ($126 - $$2,377,589) | $37,828 ($84 - $2,638,260) | $61,262 ($121 - $2,718,433) |
| **Colorectal Cancer** | N=2,407 | | | |
| n | 269 | 581 | 914 | 643 |
| Standard Cost, mean (SD) | $110,882 ($105,458) | $127,692 ($102,528) | $144,559 ($114,609) | $255,666 ($129,320) |
| Standard Cost, median (range) | $11,713 ($52 - 3,246,525) | $17,046 ($31 - $3,518,948) | $26,129 ($0 - $5,018,125) | $117,525 ($34 - $4,164,527) |
| **Esophageal Cancer** | N=377 | | | |
| n | 29 | 96 | 104 | 148 |
| Standard Cost, mean (SD) | $153,413 ($140,987) | $287,573 ($169,974) | $300,948 ($230,180) | $344,711 ($183,436) |
| Standard Cost, median (range) | $32,124 ($128 - $1,510,744) | $73,344 ($381 - $$3,193,233) | $91,739 ($84 - 4,928,730) | $146,342 ($1,334 - $3,810,669) |
| **Head and Neck Cancer** | N=728 | | | |
| n | 130 | 210 | 173 | 215 |
| Standard Cost, mean (SD) | $123,891 ($26,374) | $169,379 ($33,048) | $198,374 ($37,075) | $171,546 ($39,884) |
| Standard Cost, median (range) | $26,374 ($25 - $2,176,000) | $33,048 ($95 - $3,365,057) | $37,075 ($105 - $3,955,482) | $39,884 ($56 - $4,172,947) |
| **Kidney and Renal Pelvis Cancer** | N=300 | | | |
| n | 46 | 60 | 56 | 138 |
| Standard Cost, mean (SD) | $63,067 ($55,158) | $109,947 ($128,086) | $155,906 ($153,040) | $349,382 ($167,966) |
| Standard Cost, median (range) | $7,663 ($73 - $787,757) | $10,475 ($84 - $1,962,698) | $19,698 ($300 - $1,946,533) | $135,738 ($166 - $3,410,842) |
| **Lung Cancer** | N=3,459 | | | |
| n | 793 | 483 | 711 | 1472 |
| Standard Cost, mean (SD) | $161,116 ($145,174) | $244,234 ($161,199) | $307,472 ($253,829) | $418,591 ($193,014) |
| Standard Cost, median (range) | $25,627 ($75 - $6,519,699) | $57,929 ($98 - $5,209,832) | $106,973 ($64 - $9,738,636) | $211,005 ($20 - $7,569,157) |
| **Melanoma Cancer** | N=376 | | | |
| n | 164 | 104 | 71 | 37 |
| Standard Cost, mean (SD) | $30,264 ($25,870) | $106,574 ($136,012) | $99,904 ($62,778) | $202,591 ($108,518) |
| Standard Cost, median (range) | $5,094 ($52 - $736,586) | $9,534 ($84 - $2,679,789) | $15,592 ($263 - $1,107,409) | $57,673 ($306 - $1,337,159) |
| **Other Cancer** | N=253 | | | |
| n | 16 | 50 | 57 | 130 |
| Standard Cost, mean (SD) | $174,439 ($111,913) | $140,417 ($125,593) | $146,684 ($89,718) | $254,623 ($151,806) |
| Standard Cost, median (range) | $35,650 ($538 - $1,057,810) | $22,324 ($51 - $1,677,930) | $43,290 ($146 - $1,435,796) | $89,924 ($28 - $3,227,109) |
| **Ovarian Cancer** | N=723 | | | |
| n | 247 | 79 | 270 | 127 |
| Standard Cost, mean (SD) | $63,474 ($70,675) | $133,060 ($104,026) | $159,513 ($104,248) | $217,214 ($132,691) |
| Standard Cost, median (range) | $5,527 ($48 - $1,834,206) | $20,992 ($128 - $1,675,309) | $49,870 ($34 - $2,687,404) | $98,092 ($42 - $2,633,749) |
| **Pancreatic Cancer** | N=563 | | | |
| n | 43 | 137 | 79 | 304 |
| Standard Cost, mean (SD) | $234,982 ($131,912) | $280,526 ($143,961) | $305,273 ($138,300) | $337,379 ($170,112) |
| Standard Cost, median (range) | $76,420 ($95 - $1,674,187) | $113,197($52 - $2,756,993) | $154,224 ($253 - $2,214,351) | $158,272 ($275 - $4,360,600) |
| **Prostate Cancer** | N=2,079 | | | |
| n | 459 | 815 | 264 | 541 |
| Standard Cost, mean (SD) | $60,530 ($77,391) | $77,416 ($70,013) | $82,712 ($60,389) | $147,182 ($104,193) |
| Standard Cost, median (range) | $6,099 ($17 - $2,760,929) | $9,407 ($17 - $3,219,995) | $8,859 ($17 - $1,628,960) | $36,639 ($26 - $4,361,454) |
| **Stomach Cancer** | N=164 | | | |
| n | 17 | 42 | 37 | 68 |
| Standard Cost, mean (SD) | $87,607 ($67,492) | $271,247 ($164,954) | $266,852 ($169,148) | $296,930 ($136,134) |
| Standard Cost, median (range) | $11,146 ($293 - $621,298) | $89,265 ($212 - $2,428,985) | $76,818 ($219 - $2,018,506) | $154,017 ($1,246 - $2,278,663) |
| **Testicular Cancer** | N=199 | | | |
| n | 105 | 60 | 25 | 9 |
| Standard Cost, mean (SD) | $26,999 ($34,009) | $43,537 ($43,775) | $141,569 ($145,391) | $259,516 ($220,995) |
| Standard Cost, median (range) | $3,384 ($84 - $782,478) | $5,323 ($36 - $770,048) | $10,551 ($71 - $1,562,589) | $26,798 ($168 - $1,513,732) |
| **Thyroid Cancer** | N=809 | | | |
| n | 487 | 129 | 109 | 84 |
| Standard Cost, mean (SD) | $40,673 ($51,674) | $68,630 ($123,461) | $70,833 ($77,114) | $158,081 ($135,119) |
| Standard Cost, median (range) | $4,496 ($46 - $2,159,503) | $4,932 ($34 - $2,489,600) | $8,768 ($31 - $1,638,733) | $19,243 ($175 - $2,957,604) |
| **Ureter/Urinary Bladder Cancer** | N=331 | | | |
| n | 36 | 128 | 62 | 105 |
| Standard Cost, mean (SD) | $122,569 ($88,477) | $205,031 ($131,852) | $222,550 ($164,929) | $263,814 ($177,315) |
| Standard Cost, median (range) | $30,952 ($112 - $1,388,087) | $47,366 ($24 - $2,697,274) | $47,593 ($179 - $2,090,114) | $71,723 ($131 - $3,497,726) |
| **Vaginal Cancer** | N=124 | | | |
| n | 61 | 23 | 27 | 13 |
| Standard Cost, mean (SD) | $67,643 ($49,987) | $77,472 ($53,816) | $200,055 ($114,077) | $333,015 ($199,894) |
| Standard Cost, median (range) | $14,396 ($30 - $929,505) | $17,054 ($365 - $590,416) | $69,145 ($126 - $1,396,686) | $104,209 ($2,393 - $1,415,671) |

**Additional Table 3a. Breast Cancer Member Characteristics by Stage, Diagnosed 2016-2020 Comparison with USCS Data as of 2019**

| Stage | **I** | **II** | **III** | **IV** | **USCS**^c^ |
| --- | --- | --- | --- | --- | --- |
| n (%)^a, b^ | 51.2% | 34.1% | 8.1% | 6.6% | Localized: 66.0%  Regional: 25.8%  Distant: 5.8%  Unstaged: 2.4% |
| **Age**, mean (SD), years^b^ | 59.7 (11.5) | 57.4 (12.4) | 56.4 (12.8) | 57.5 (12.9) | 0-39: 3.2%  40-49: 11.1%  50-59: 21.3%  60-69 28.3%  70-79: 24.1%  80+: 12.0% |
| **Female Gender**, n (%)^b^ | 99.7% | 99.3% | 99.4% | 99.1% | 100% |

SD, standard deviation; USCS, United States Cancer Statistics

^a^ Percentages may not total to 100% due to rounding

^b^ Demographics were calculated at the time of cancer diagnosis

^c^ Only includes female breast cancer patients; Stage captured USCS 2015-2019; Age and gender reflects USCS 5-year limited duration on January 1, 2019

**Additional Table 3b. Cervical Cancer Member Characteristics by Stage, Diagnosed 2016-2020 Comparison with USCS Data as of 2019**

| Stage | **I** | **II** | **III** | **IV** | **USCS**^c^ |
| --- | --- | --- | --- | --- | --- |
| n (%)^a, b^ | 69.7% | 10.6% | 11.5% | 8.2% | Localized: 44.2%  Regional: 34.4%  Distant: 15.0%  Unstaged: 6.5% |
| **Age**, mean (SD), years^b^ | 58.6 (12.3) | 57.5 (12.1) | 60.4 (12.2) | 59.9 (12.7) | 0-29: 2.9%  30-39: 20.2%  40-49: 26.3%  50-59: 22.3%  60-69: 17.0%  70+: 11.4% |
| **Female Gender**, n (%)^b^ | 100.0% | 100.0% | 100.0% | 100.0% | 100.0% |

SD, standard deviation; USCS, United States Cancer Statistics

^a^ Percentages may not total to 100% due to rounding

^b^ Demographics were calculated at the time of cancer diagnosis

^c^ Stage captured USCS 2015-2019; Age and gender reflects USCS 5-year limited duration on January 1, 2019

**Additional Table 3c. Colorectal Cancer Member Characteristics by Stage, Diagnosed 2016-2020 Comparison with USCS Data as of 2019**

| Stage | **I** | **II** | **III** | **IV** | **USCS**^c^ |
| --- | --- | --- | --- | --- | --- |
| n (%)^a, b^ | 11.2% | 24.1% | 38.0% | 26.7% | Localized: 34.8%  Regional: 37.3%  Distant: 21.5%  Unstaged: 6.4% |
| **Age**, mean (SD), years^b^ | 63.2 (12.5) | 62.2 (13.8) | 61.0 (13.0) | 58.8 (13.2) | 0-49: 10.8%  50-59: 20.3%  60-69: 27.0%  70-79: 24.3%  80+: 17.6% |
| **Female Gender**, n (%)^b^ | 49.1% | 46.0% | 47.5% | 46.4% | 47.6% |

SD, standard deviation; USCS, United States Cancer Statistics

^a^ Percentages may not total to 100% due to rounding

^b^ Demographics were calculated at the time of cancer diagnosis

^c^ Stage captured USCS 2015-2019; Age and gender reflects USCS 5-year limited duration on January 1, 2019

**Additional Table 3d. Lung Cancer Member Characteristics by Stage, Diagnosed 2016-2020 Comparison with USCS Data as of 2019**

| Stage | **I** | **II** | **III** | **IV** | **USCS**^c^ |
| --- | --- | --- | --- | --- | --- |
| n (%)^a, b^ | 22.9% | 14.0% | 20.6% | 42.6% | Localized: 26.2%  Regional: 22.7%  Distant: 46.4%  Unstaged: 4.7% |
| **Age**, mean (SD), years^b^ | 68.6 (9.9) | 66.9 (10.1) | 66.7 (9.9) | 65.1 (10.6) | 0-49: 2.7%  50-59: 12.2%  60-69: 30.3%  70-79: 36.7%  80+: 18.1% |
| **Female Gender**, n (%)^b^ | 59.5% | 50.9% | 49.9% | 50.7% | 53.6% |

SD, standard deviation; USCS, United States Cancer Statistics

^a^ Percentages may not total to 100% due to rounding

^b^ Demographics were calculated at the time of cancer diagnosis

^c^ Stage captured USCS 2015-2019; Age and gender reflects USCS 5-year limited duration on January 1, 2019

**Additional Table 3e. Ovarian Cancer Member Characteristics by Stage, Diagnosed 2016-2020 Comparison with USCS Data as of 2019**

| Stage | **I** | **II** | **III** | **IV** | **USCS**^c^ |
| --- | --- | --- | --- | --- | --- |
| n (%)^a, b^ | 34.2% | 10.9% | 37.3% | 17.6% | Localized: 20.8%  Regional: 22.9%  Distant: 50.3%  Unstaged: 6.0% |
| **Age**, mean (SD), years^b^ | 53.5 (14.6) | 58.5 (12.1) | 59.6 (13.3) | 61.5 (11.3) | 0-39: 9.1%  40-49: 11.2%  50-59: 23.2%  60-69: 27.6%  70-79: 20.1%  80+: 8.7% |
| **Female Gender**, n (%)^b^ | 100.0% | 100.0% | 100.0% | 100.0% | 100.0% |

SD, standard deviation; USCS, United States Cancer Statistics

^a^ Percentages may not total to 100% due to rounding

^b^ Demographics were calculated at the time of cancer diagnosis

^c^ Stage captured USCS 2015-2019; Age and gender reflects USCS 5-year limited duration on January 1, 2019

**Additional Table 3f. Prostate Cancer Member Characteristics by Stage, Diagnosed 2016-2020 Comparison with USCS Data as of 2019**

| Stage | **I** | **II** | **III** | **IV** | **USCS**^c^ |
| --- | --- | --- | --- | --- | --- |
| n (%)^a, b^ | 22.1% | 39.2% | 12.7% | 26.0% | Localized: 70.6%  Regional: 13.5%  Distant: 7.6%  Unstaged: 8.3% |
| **Age**, mean (SD), years^b^ | 62.8 (8.0) | 66.2 (8.3) | 65.8 (9.6) | 68.1 (10.4) | 0-49: 1.0%  50-59: 13.5%  60-69: 40.2%  70-79: 35.4%  80+: 10.0% |
| **Female Gender**, n (%)^b^ | 0.0% | 0.0% | 0.0% | 0.0% | 0.0% |

SD, standard deviation; USCS, United States Cancer Statistics

^a^ Percentages may not total to 100% due to rounding

^b^ Demographics were calculated at the time of cancer diagnosis

^c^ Stage captured USCS 2015-2019; Age and gender reflects USCS 5-year limited duration on January 1, 2019

**Additional Table 4. Generalized Linear Regression Analysis on Monthly Treatment Costs During Year 1 by Stage at Diagnosis^a^**

| **Variable** | **Mean Monthly Cost in Year 1 Per Patient**^b^ | | |
| --- | --- | --- | --- |
|  | Coefficient^c^ | Std. Error | P-value |
| **Breast Cancer** | | | |
| Stage II Diagnosis | 4,267 | 184 | <.0001 |
| Stage III Diagnosis | 7,286 | 316 | <.0001 |
| Stage IV Diagnosis | 8,905 | 317 | <.0001 |
| **Cervical Cancer** | | | |
| Stage II Diagnosis | 7,210 | 575 | <.0001 |
| Stage III Diagnosis | 7,503 | 607 | <.0001 |
| Stage IV Diagnosis | 8,229 | 705 | <.0001 |
| **Colorectal Cancer** | | | |
| Stage II Diagnosis | 2,605 | 866 | 0.003 |
| Stage III Diagnosis | 5,785 | 828 | <.0001 |
| Stage IV Diagnosis | 13,523 | 887 | <.0001 |
| **Lung Cancer** | | | |
| Stage II Diagnosis | 8,222 | 1,163 | <.0001 |
| Stage III Diagnosis | 12,125 | 1,075 | <.0001 |
| Stage IV Diagnosis | 19,036 | 928 | <.0001 |
| **Ovarian Cancer** | | | |
| Stage II Diagnosis | 5,529 | 1,405 | <.0001 |
| Stage III Diagnosis | 5,200 | 974 | <.0001 |
| Stage IV Diagnosis | 9,531 | 1163 | <.0001 |
| **Prostate Cancer** | | | |
| Stage II Diagnosis | 687 | 438 | 0.10 |
| Stage III Diagnosis | 571 | 586 | 0.30 |
| Stage IV Diagnosis | 4,916 | 479 | <.0001 |
| ^a^Detailed regression results by cancer type are available from the authors upon request  ^b^Effects are shown as adjusted for sex, race, age group (under 65 vs. 65 and older), Charlson Comorbidity Index, insurance type, region, and month 1-12 dummy variables; Costs are in 2020 US dollars  ^b^Reported coefficients are relative to the base group: mean total monthly costs for patients with Stage I diagnosis | | | |

**Additional Figure 1a. Breast Cancer Mean Cost by Stage at Diagnosis, Cumulative Through Year 4 Post Diagnosis**

**Additional Figure 1b. Cervical Cancer Mean Cost by Stage at Diagnosis, Cumulative Through Year 4 Post Diagnosis**

**Additional Figure 1c. Colorectal Cancer Mean Cost by Stage at Diagnosis, Cumulative Through Year 4 Post Diagnosis**

**Additional Figure 1d. Lung Cancer Mean Cost by Stage at Diagnosis, Cumulative Through Year 4 Post Diagnosis**

**Additional Figure 1e. Ovarian Cancer Mean Cost by Stage at Diagnosis, Cumulative Through Year 4 Post Diagnosis**

**Additional Figure 1f. Prostate Cancer Mean Cost by Stage at Diagnosis, Cumulative Through Year 4 Post Diagnosis**

**Additional Figure 2. Mean Cancer-specific Cost by Cancer and Stage, Year 1 Post Diagnosis: 1-6 months and 7-12 months**

**Prostate Cancer**

**Ovarian Cancer**

**Lung Cancer**

7-12 mo

1-6 mo

7-12 mo

1-6 mo

7-12 mo

1-6 mo

**Additional Figure 3a. Breast Cancer Mean Cancer-specific Cost by Stage at Diagnosis, Year 1-4 Post Diagnosis**

**Additional Figure 3b. Cervical Cancer Mean Cancer-specific Cost by Stage at Diagnosis, Year 1-4 Post Diagnosis**

**Additional Figure 3c. Colorectal Cancer Mean Cancer-specific Cost by Stage at Diagnosis, Year 1-4 Post Diagnosis**

**Additional Figure 3d. Lung Cancer Mean Cancer-specific Cost by Stage at Diagnosis, Year 1-4 Post Diagnosis**

**Additional Figure 3e. Ovarian Cancer Mean Cancer-specific Cost by Stage at Diagnosis, Year 1-4 Post Diagnosis**

**Additional Figure 3f. Prostate Cancer Mean Cancer-specific Cost by Stage at Diagnosis, Year 1-4 Post Diagnosis**

**Additional Figure 4a. Breast Cancer Mean Cancer-specific Cost by Stage at Diagnosis, Cumulative Through Year 4 Post Diagnosis**

**Additional Figure 4b. Cervical Cancer Mean Cancer-Specific Cost by Stage at Diagnosis, Cumulative Through Year 4 Post Diagnosis**

**Additional Figure 4c. Colorectal Cancer Mean Cancer-Specific Cost by Stage at Diagnosis, Cumulative Through Year 4 Post Diagnosis**

**Additional Figure 4d. Lung Cancer Mean Cancer-specific Cost by Stage at Diagnosis, Cumulative Through Year 4 Post Diagnosis**

**Additional Figure 4e. Ovarian Cancer Mean Cancer-specific Cost by Stage at Diagnosis, Cumulative Through Year 4 Post Diagnosis**

**Additional Figure 4f. Prostate Cancer Mean Cancer-specific Cost by Stage at Diagnosis, Cumulative Through Year 4 Post Diagnosis**
